# ‘The Excellent Evaluators’: Co‐Producing an Evaluation of the Hertfordshire Young People's Research Advisory Group (Herts YPAG)

**DOI:** 10.1111/hex.70949

**Published:** 2026-10-30

**Authors:** Lauren Denyer, Helen Abnett, Benita Dijan, Zuyi Su, Jacob Wright, Louca‐Mai Brady

**Affiliations:** ^1^ Centre for Research in Public Health and Community Care (CRIPACC), School of Health, Medicine and Life Sciences University of Hertfordshire Hatfield UK

**Keywords:** co‐production, evaluation, health and social care research, PPIE, YPAG

## Abstract

**Introduction:**

Patient and public involvement (PPI) is an established way to improve the quality and impact of health and care research. All children have a right to have a say in decisions that affect them, and Young People's Research Advisory Groups are now an established approach for enabling young people's involvement in research. However, there is limited evidence considering how to evaluate such groups. In this article, we describe the process of co‐producing an evaluation of the Hertfordshire Young Person's Advisory Group (YPAG) hosted by the University of Hertfordshire, UK (UH) in collaboration with young group members.

**Methods:**

We provide an in‐depth account of how the team worked together, utilising a creative and collaborative approach to design the evaluation.

**Results:**

The findings show that researchers, young people and parents/carers value children and young people's involvement in research. Findings also demonstrate the benefits for young people of being a member of the group, such as the development of new skills.

**Conclusion:**

Co‐production of this evaluation was enabled through the adoption and practice of an approach that combined flexibility and creativity with appropriate resourcing. However, we also share the challenges with our approach, including complexities around data sharing. Nevertheless, we argue that co‐producing the evaluation enabled us to learn about the strengths of the group, while also highlighting helpful learning for the future planning of the group, and contributing to evidence on the importance of evaluating PPI.

**Patient and Public Involvement:**

Three young people (two aged 17; one aged 12) were equal members of the five‐person evaluation team, involved throughout alongside two UH researchers. During the 6‐month evaluation process, this team met 12 times (online or face‐to‐face). Additional discussions were conducted over email.

## 1 Introduction

Public involvement is defined by the National Institute for Health and Care Research (NIHR) as ‘research being carried out “with” or “by” members of the public rather than “to”, “about” or “for” them… an active partnership between patients, carers and members of the public’ [1]. Public involvement is an important component of health and care research in the United Kingdom [2, 3, 4] and internationally [5, 6]. There is growing evidence of the importance of involving children and young people in research [7, 8, 9], and there are a range of benefits of doing this. This includes improving the relevance of research, methods of data collection and dissemination, and providing the opportunity for young people to help to instigate change, develop useful skills and improve their own confidence and self‐esteem [10, 11, 12].

Alongside the arguments for the impact and benefits of young people's involvement, there is also a moral argument: as outlined in the United Nations Convention on the Rights of the Child [13], children and young people have a right to information and to have a say in decisions that affect them, including research [7]. A rights‐based approach recognises that children and young people are not just passive sources of data but can and should have active agency in knowledge creation [14]. In addition to these consequentialist (public involvement of young people improves the quality and impact of research and has benefits for young people) and moral (rights to meaningful involvement) arguments, Warraitch et al. [15] suggest a third political and pragmatic argument: that involvement provides a means to bridge the democratic gap and provide accountability by providing young people with a voice in publicly funded research. However, how and when children and young people are involved in health and care research in the United Kingdom is still largely determined and controlled by adults [14]. Their involvement is also insufficiently documented and evaluated compared to adult PPI [16].

### 1.1 Co‐Production and Creative Methods

Public involvement can take many forms including members of the public acting as ‘joint grant holders or co‐applicants on a research project, identifying research priorities, as members of a project advisory or steering group’, contributing to the development of research tools and materials, data collection, analysis, reporting and dissemination [1]. In co‐production, researchers and members of the public work together to share responsibility for a research project, including the generation of knowledge [17, 18]. This includes both co‐creation (‘collaboration and creative problem solving between stakeholders at all stages of a piece of work’) and codesign (‘active collaboration between stakeholders to design solutions to a set problem’), and ‘encompasses all co‐approaches’ [19, p. 2]. However, co‐production with children and young people is complicated due to unequal power dynamics that exist between adults and children [20] and requires a lot of time and resources, including for data analysis [21, 22]. Creative or arts‐based research methods (e.g., artwork, drama, mapping or Lego) are typically more engaging than traditional approaches and can be used to facilitate conversation and reflection [23, 24]. However, there is often confusion between the use of creative participatory approaches and qualitative methodology [25, 26], and as Phillips et al. [27] note, there is a ‘dearth of detailed analyses’ of the potentials and challenges of creative and arts‐based research methods.

### 1.2 YPAGs and the Importance of Evaluation

The Lundy model of participation [28] suggests four elements are vital for the meaningful participation of children and young people. These four elements are space: young people are given the opportunity to express their views; voice: young people are facilitated to express their views; audience: their views are heard, and influence: their views are acted upon. A common approach for children and young people's involvement in UK health and care research is the Young Person's Advisory Group (YPAG): a forum in which researchers can present their ideas and offer a space for children and young people to provide guidance and insight into research activities [29]. A YPAG may be established for a specific research study or may also be hosted by an institution in which young people provide feedback to lots of different researchers. The YPAG model allows a group of children and young people to develop an understanding of research issues and processes through practical experience and training. YPAGs hosted by institutions, as opposed to those which are study/project‐specific, also provide a convenient way for researchers to get input from children and young people on a one‐off or ongoing basis by attending YPAG meetings supported by experienced facilitators [7, 16]. In contributing to research, children and young people can take a consultative role through improving information sheets or take more of a collaborative role in shaping research priorities or developing tools alongside researchers [30].

Regular evaluation is important for the ongoing monitoring and development of young people's involvement groups, including YPAGs [30], as well as identifying good practice and opportunities for learning [31]. Yet, there is a significant research gap in involving children and young people in research and evaluating this involvement [32]. Building on the evaluation of the eyeYPAG based at Moorfields Hospital [16], this evaluation seeks to contribute to closing this evidence gap, by exploring the value of engagement with the Herts YPAG, both for researchers and for YPAG members themselves. This article and the report and summary on which it draws [33] have been co‐authored by young co‐evaluators from the Herts YPAG (alongside group facilitators) who helped plan the paper and reviewed and contributed to drafts. This paper describes how we co‐produced the evaluation and outlines the findings.

### 1.3 The Herts YPAG

The Herts YPAG is a group of young people aged 10–18 living in Hertfordshire, run by the Centre for Research in Public Health and Community Care (CRIPACC) in the School of Health, Medicine and Life Sciences at the University of Hertfordshire, UK (UH). The purpose of the group—as the YPAG members themselves define—is to ‘learn about research and work with researchers to help them to plan and do research about children and young people's health, care, family lives and the communities we live in’ [34]. The group provide support to researchers across the research process, including designing research, developing materials (e.g., information sheets and consent forms), analysis, reporting and dissemination. They primarily provide input for researchers at UH and the NIHR Applied Research Collaboration East of England, as well as researchers and policymakers from other academic institutions and voluntary sector or government organisations. Herts YPAG members have also co‐facilitated training, presented at seminars and conferences, recorded a short podcast series and co‐created a film about the group. The group is part of GenerationR, a UK‐wide network of YPAGs across the United Kingdom supporting the involvement of children and young people in health and care research, and the European Young Persons' Advocacy Group (eYPAGnet), a virtual consortium of YPAGs across Europe.

The Herts YPAG restarted in spring 2022, after the original group came to an end during the COVID‐19 pandemic as the existing members grew up and moved onto university. At the time of conducting the evaluation, the Herts YPAG consisted of 13 young people, aged 10–18 years old.

### 1.4 Aim of the Evaluation

This evaluation was a co‐designed, co‐led reflective process evaluation, focusing on the operation of the Herts YPAG, and whether and how its planned outcomes are being achieved [35]. Building on an earlier co‐produced evaluation of another GenR YPAG [16], the evaluation team collaboratively developed the evaluation questions:

1. How has being a member of the YPAG impacted YPAG members themselves?
2. How has working with the YPAG impacted research/researchers?
3. How can the YPAG be improved for the future?

### 1.5 The Evaluation Team: ‘The Excellent Evaluators’

With the support of some internal ‘participatory research’ funding, the Herts YPAG facilitators (L.D., H.A. and L.‐M.B.) developed initial plans for the evaluation and asked for Herts YPAG members to volunteer if they wanted to take part at an in‐person meeting in March 2025. Three YPAG members, and co‐authors of this paper, volunteered to be part of the evaluation team and chose the name ‘The Excellent Evaluators’.

## 2 Our Approach and Methodology

### 2.1 How the ‘Excellent Evaluators’ Team Worked Together

Informed by co‐production principles [17] we sought to make the evaluation process as collaborative, reflexive and flexible as possible, with the evaluation team working together, primarily online over Zoom and email. The young co‐evaluators were given opportunities to be involved throughout the process, with different elements led by different members of the team depending on interests and availability (the young co‐evaluators all had to balance their involvement with school and other commitments).

During meetings, we used games and reflective tools to check‐in with each other, highlight any concerns and celebrate our achievements. In total, we held 11 online Excellent Evaluators team meetings, as well as one in‐person meeting, between April and October 2025 (see Table 1). During our third meeting, we developed an Excellent Evaluators' Team Agreement (Appendix 1), which outlined our collective roles and responsibilities, including a commitment to the values of the evaluation:

We are all collectively responsible for the evaluation. We will all listen to each other's opinions and try to take decisions collectively.

**Table 1:** Excellent evaluators' team meetings and activities.

Meeting number	Date	Meeting purpose	Meeting location
1	01/04/2025	Introduction to evaluation	Zoom
2	15/04/2025	Reviewing and agreeing research plan & ideas and ethics	Zoom
3	06/05/2025	Team agreement; introduction to secondary data analysis	Zoom
4	13/05/2025	Conducting secondary data analysis	Zoom
5	20/05/2025	Planning and training for the focus groups	Zoom
6	24/05/2025	Focus groups; analysis of creative outputs from the YPAG members' focus group	In‐person
7	09/06/2025	Interview 1	Zoom
8	24/06/2025	Interview 2	Zoom
9	08/07/2025	Thematic analysis training; analysis of focus group and interview transcripts	Zoom
10	14/07/2025	Analysis of focus group and interview transcripts; co‐writing the evaluation blog post [36]	Zoom
11	30/09/2025	Reviewing outputs (this article; a public facing report) [33]	Zoom
12	22/09/2025	Final review of report & summary	Zoom

The group worked collaboratively to design each stage of the evaluation. The young co‐evaluators were given research methods training by the adult researchers (L.D. and H.A.), including on writing research questions, ethics, focus group facilitation and data analysis. We kept a record of how many hours that the young co‐evaluators worked on the project so they could be paid for their time (an hourly rate) at the end of the evaluation.

### 2.2 Developing the Research Questions

During Meeting 1, we explored the aims of the evaluation and what we wanted to find out. Through this discussion we identified two key areas of interest: the experience of YPAG members, and the impact of working with the group on research and researchers. This discussion was summarised by an adult researcher in a PowerPoint slide (see Figure 1) and shared with the team at the following meeting, where we agreed a third area of interest: the future of the YPAG. Based on these discussions, we collectively agreed our research questions.

**Figure 1:**
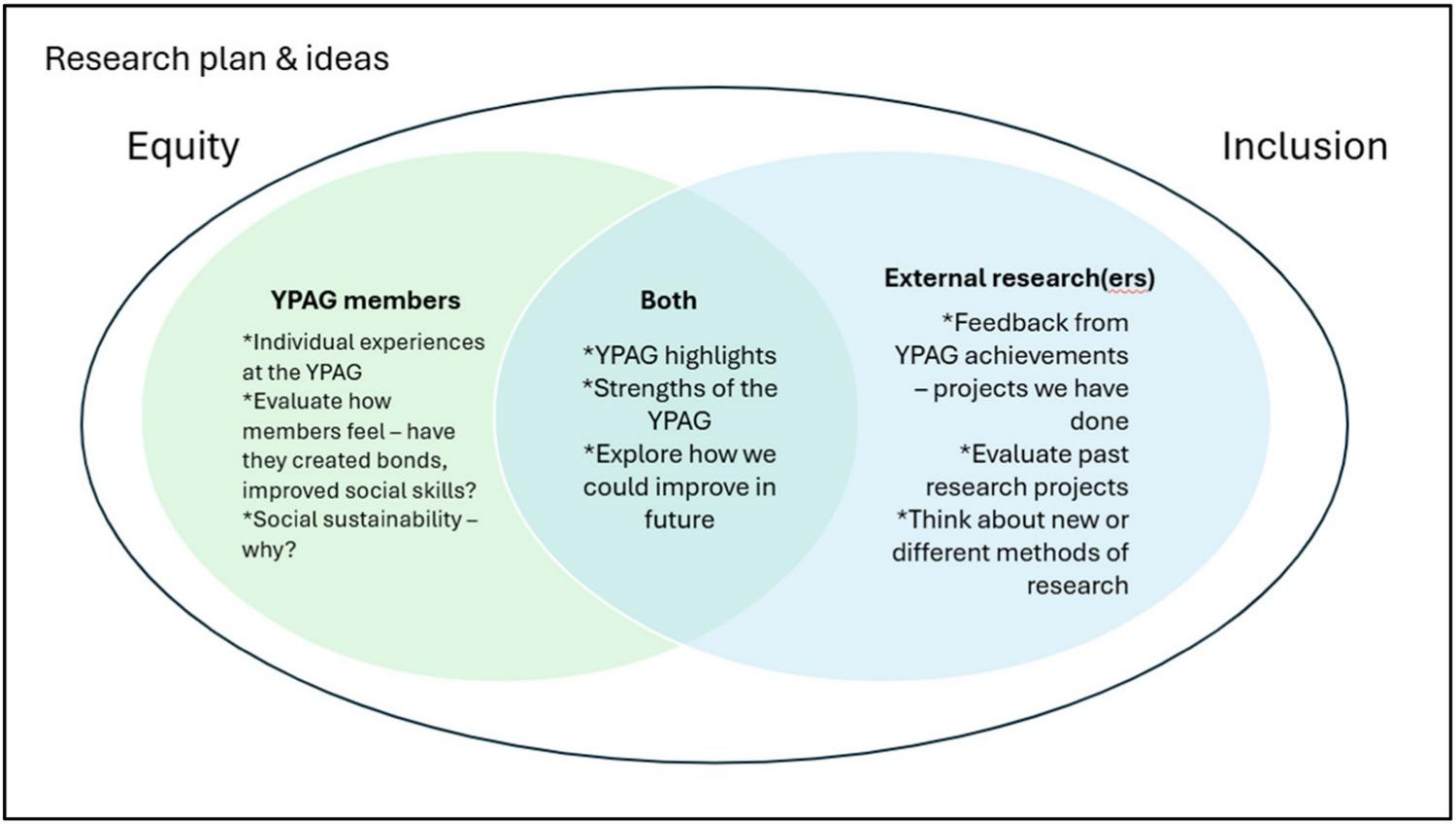
Summary for our research questions discussion.

## 3 Methods

### 3.1 Data Collection

The Excellent Evaluators team agreed on a multi‐modal approach to data collection and analysis, involving focus groups, interviews, surveys and secondary analysis of existing data. We chose this approach to allow for different methods for participants to share their experiences [37]. The topic guides and surveys were adapted for different stakeholder groups to reflect their distinct experiences and perspectives (see Appendix for examples).

#### 3.1.1 Focus Groups

Two in‐person focus groups were held in May 2025 facilitated by young co‐evaluators with support from the adult researchers. The groups were audio‐recorded and transcribed for analysis.

1. Focus Group 1 was led by two of the young co‐evaluators and six Herts YPAG members attended. The topic guide was created specifically for this focus group and used creative activities for qualitative data collection alongside discussion to help make the focus group more accessible and engaging. One of the creative qualitative methods asked YPAG members to individually write or draw on a shield template to describe what skills they felt they had gained from being a member of the YPAG. These creative activities served both to facilitate participation, reflection and to generate qualitative data. The creative outputs produced by participants were retained as evaluation data and analysed alongside the other data collected as described below.
2. Focus Group 2 was led by two of the young co‐evaluators, with the three adult facilitators (L.D., H.A. and L.‐M.B.) as participants. As with Brady et al. [16], this provided an interesting opportunity to reverse the normal adult/child and facilitator/YPAG member power dynamic. The focus group topic guide questions were developed independently by the young co‐evaluators with no input from the adult researchers.

#### 3.1.2 Interviews

Two researchers who were external to the Herts YPAG and had ongoing relationships with the group (through often presenting at group meetings) agreed to be interviewed as part of the evaluation. Before conducting the interviews, the young co‐evaluators received training from the adult researchers on active listening and interview and focus group facilitation approaches, drawing on the ‘Young Researcher Toolkit’ [38]. Examples of possible interview questions were generated during an Excellent Evaluators online meeting. These questions, alongside some additional suggestions by the adult researchers, were then included in the final interview schedule. These in‐depth semi‐structured interviews were then conducted by the young co‐evaluators (Interview 1 was conducted by one young co‐evaluator, and Interview 2 was conducted by all three young co‐evaluators) and took place online via Zoom. The interviews were recorded and transcribed.

#### 3.1.3 Surveys

Three surveys were conducted and shared using Microsoft Forms: one for YPAG members unable to attend the in‐person focus group (*n* = 4; 100% response rate); one for researchers who had worked with the group (responses = 7; 33% response rate); and one for parents and carers of YPAG members (*n* = 8; 62% response rate). The YPAG member survey included the same questions as the focus group but without the creative activities.

#### 3.1.4 Secondary Data (Researcher Feedback Forms)

We also reviewed researcher feedback forms (*n* = 16) which had been collected after each meeting. The forms contain information about their experience of attending and presenting at the YPAG. The data collected are both quantitative and qualitative (Table 2).

**Table 2:** Number of participants across different methods.

Data collection method	Number of participants
**Focus groups**	
Focus Group 1	6 YPAG members
Focus Group 2	3 group facilitators
**Interviews**	
Interview 1	1 researcher
Interview 2	1 researcher
**Surveys**	
YPAG member survey	4 YPAG members
Parent/carer survey	8
Researcher survey	7
**Secondary data**	
Researcher feedback forms	16

### 3.2 Data Analysis

Data analysis was an iterative process, involving several different collaborative methods.

#### 3.2.1 Quantitative Data

The quantitative (scale‐based) data consisted of secondary data from the researcher feedback forms. These data were summarised by an adult researcher into tables and charts in Excel and then analysed descriptively and collaboratively during Zoom meetings.

#### 3.2.2 Creative, Paper‐Based Outputs

As discussed above, during Focus Group 1, paper‐based creative qualitative data were produced (Figure 3). During the in‐person meeting (6) after the focus groups, J.W. led the initial analysis of this data, primarily using a young people‐friendly version of quantitative content analysis [39].

#### 3.2.3 Data Analysis of Surveys and Transcripts

These data consisted of transcripts of the two focus groups and two interviews, as well as qualitative responses to survey questions. Transcription was conducted by an external company who signed a transcriber confidentiality agreement. Qualitative data were analysed using a deductive thematic analysis approach [40, 41, 42], adapted to be accessible for young people as outlined below.

##### 3.2.3.1 Phase 1: Data Familiarisation

Data‐sharing constraints meant that full transcripts could not be shared with the young co‐evaluators. This restriction and young co‐evaluators' availability meant that the adult researchers conducted the initial data familiarisation exercise with the young co‐evaluators' agreement.

##### 3.2.3.2 Phase 2: Generating Initial Codes

The adult researchers selected and prepared an illustrative range of data extracts from focus groups and interview transcriptions. These were shared with the young co‐evaluators during meeting nine, and ideas for codes were collaboratively developed through group discussion. This informed a coding framework which was applied to the full focus group and interview transcriptions by the adult researchers. During this process, some additional codes were identified, which were then discussed with the young co‐evaluators at the following meeting (see next phase) (see example in Figure 2).

**Figure 2:**
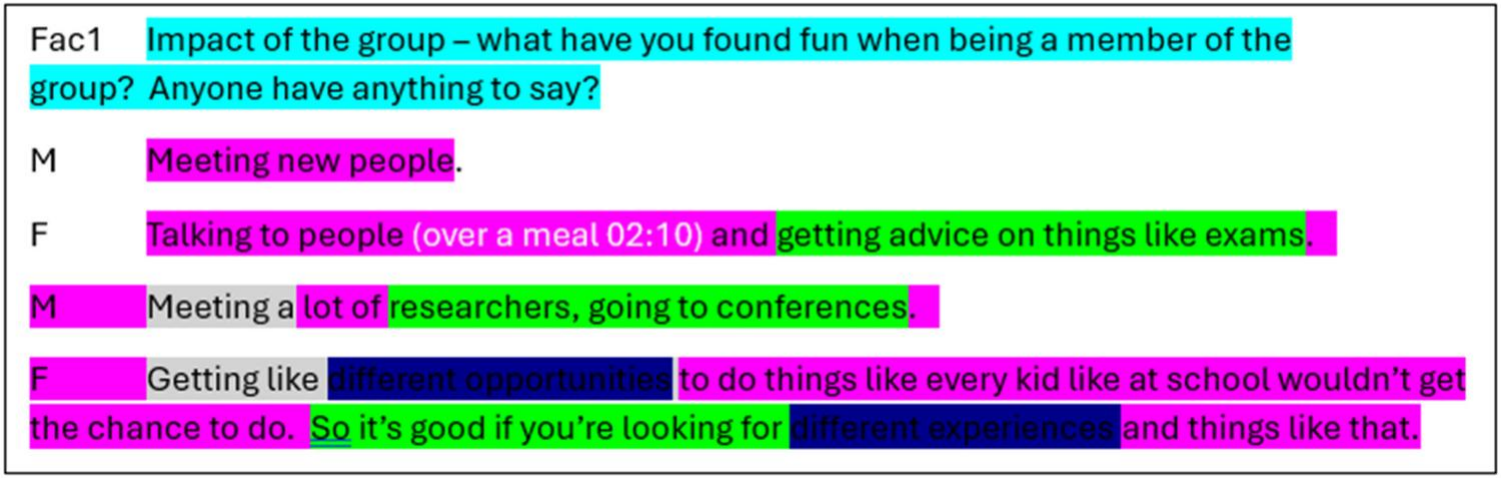
Screenshot of the initial coding of a transcript during an Excellent Evaluators meeting; note the above is the final ‘layer’ some phrases were assigned to multiple codes. Key: light blue = question, dark blue = doing something different, pink = social, grey = new experiences and green = education/learning.

##### 3.2.3.3 Phase 3: Searching for Themes and Phase 4: Reviewing and Refining Themes

During the next meeting (10)—the evaluation team discussed the codes generated from our data and generated overarching themes which related back to the original evaluation questions.

##### 3.2.3.4 Phase 5: Defining and Naming Themes and Phase 6: Writing‐Up

Defining and writing‐up the themes was a process that the team agreed would be led by the adult researchers. The draft report and a draft of this article were then shared with the Excellent Evaluators team, for discussion, review and revision.

### 3.3 Ethics

This study received ethical approval from the University of Hertfordshire Health, Science, Engineering and Technology Ethics Committee with Delegated Authority, Protocol number 1007 2025 May HSET. All participants were provided with information about the research and gave their consent (or, for those under 16, asset with their parents or carers providing consent) to participate in the research.

## 4 Evaluation Findings

This section outlines our main findings, under three main themes: the value of listening to children and young people; challenges and concerns, including tokenism; planning and working with children and young people.

### 4.1 Theme 1: The Value of Listening to Children and Young People

Both YPAG members and researchers emphasised the value of listening to children and young people, ensuring their views and ideas inform the design and delivery of health and social care research. Within our evaluation, researchers described how the input of YPAG members had influenced research design:

Not only do children and young people have the right to be consulted about matters that affect them, but the research is likely to be improved through their involvement, including in the design at an early stage of development.

(Researcher feedback form)

Most researchers who attended meetings to work with the YPAG (13/16) engaged with the group in the early stages of their research (prior to obtaining funding or ethics approval) and were focused on feedback or getting YPAG members' ‘perspective’ on tools or ideas. One researcher consulted the YPAG during data collection, and two others used their sessions to think with the group about ideas for dissemination. All researchers reported that input from the YPAG informed the development of their research project or proposal.

YPAG members, and their parents and carers, also demonstrated the impact that membership of the group had on the YPAG members themselves. They reported that engagement in the YPAG had improved members' confidence, enabled them to build new skills and exposed them to new areas of knowledge and learning. This includes supporting young people to exercise their right to be heard:

[YPAG member] realises what he has to say is important and he is heard’ (Parent and carer survey response), and to build longer term capabilities: ‘It's shaping his future too…he now has the confidence to put himself forward for other activities with YPAG but also outside YPAG.

(Parent and carer survey response)

One YPAG member clearly articulated the mutually reinforcing nature of these values, recognising that YPAG provided an opportunity for young people to build both their skills and expertise, through supporting improvements in the research process:

[The] YPAG is a great opportunity for young people to gain valuable experience, make a real impact in research and healthcare, and ensure youth voices shape important decisions.

(YPAG member survey response)

Young people and researchers both highlighted the importance of the non‐research focused social relationship‐building in generating trust and fostering a sense of community. This included valuing eating together, icebreakers and time set aside in meetings for creative expression and play. The importance of good relationships with and between young people, facilitators and researchers was recognised by all participants as necessary for enhancing young people's confidence in giving useful feedback to researchers, and motivation (and therefore, potentially, retention) among YPAG members. This was also linked to the value placed on the YPAG being an inclusive and accessible space:

The staff have made adaptations to help him stay engaged online…[and] will always try and find a way to help and they get the balance right between fun and work/learning.

(Parent and carer survey response)

This balance—between ‘fun’, learning and valuing the YPAG as a space to support both the development of research and the YPAG members themselves—was identified as key to the positive outcomes found in our evaluation.

Key concepts identified by YPAG members in the eight shields completed during the focus group (see example Figure 3) included: research skills, confidence, collaboration and communication, as well as friendship and fun.

**Figure 3:**
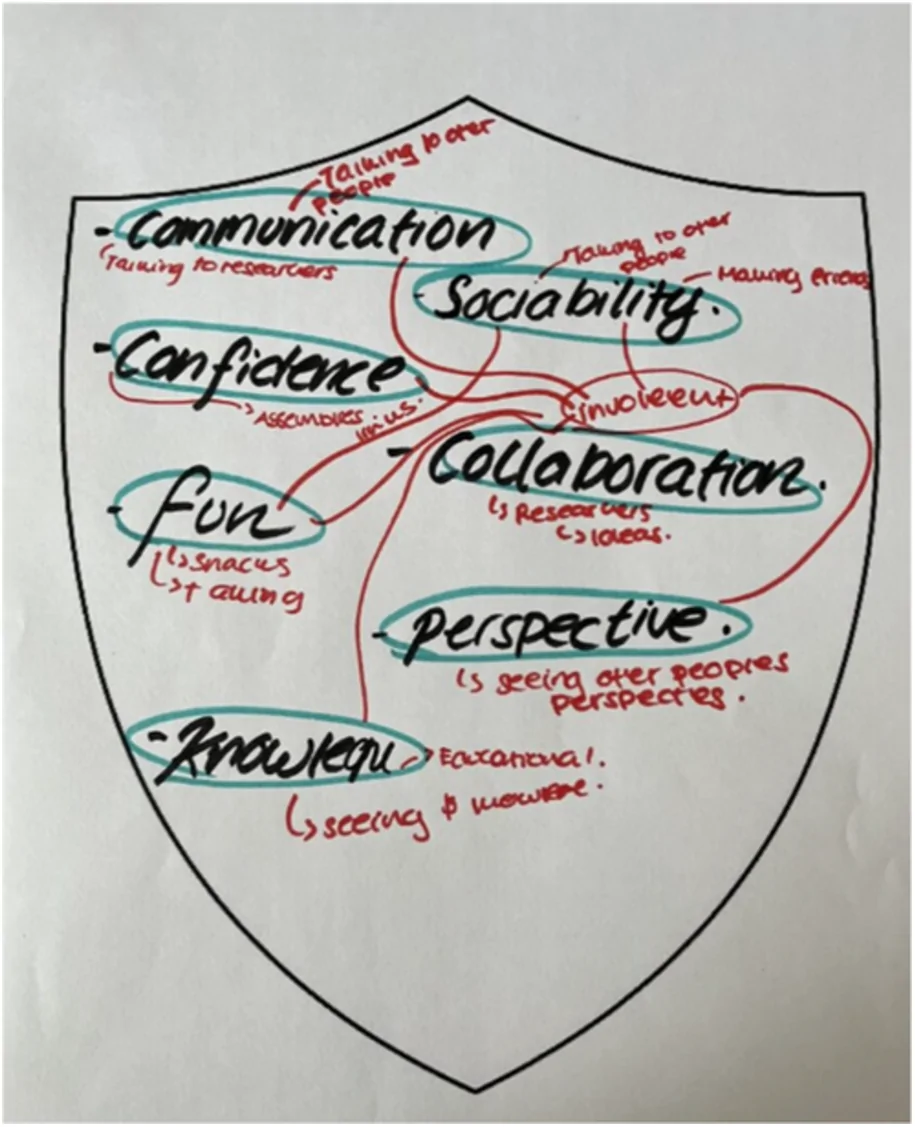
Example of a shield produced during the YPAG members' focus group.

### 4.2 Theme 2: Challenges and Concerns, Including Tokenism

YPAG members, parents and carers, and YPAG staff facilitators all raised questions about the risks of tokenism and ‘tick box’ involvement. It was suggested that some researchers may be engaging with the YPAG to fulfil funder or ethics committee requirements, rather than because of a commitment to meaningful public involvement:

Some researchers just seem to want to get young people involved so they can tick it off on the list or mention it for the funding application or something like that.

(YPAG member)

YPAG members reflected that they felt this occurred particularly when they were not given any information by researchers about the impact of their contributions:

some researchers…they just leave us [after a meeting with no feedback]…and it's like ‘okay what have I done to benefit you?’

(YPAG member)

Practical issues around the administration and organisation of meetings were raised by YPAG facilitators, and parents and carers. This includes challenges around creating a balance in the frequency, format and duration of meetings. YPAG adult facilitators described the level of logistical and administrative work involved in supporting the YPAG. The increasing number of researchers interested in working with the YPAG limited time for in‐depth engagement and researcher feedback, as well as timely opportunities for researchers to work with the group.

### 4.3 Theme 3: Planning and Working With Children and Young People

Our third theme centres around the importance of recognising that working with children and young people is different to working with adults. Researchers highlighted the importance of preparing for their sessions, including spending time planning activities and talking to YPAG facilitators in advance of the session.

It was really helpful to be able to meet with you to plan the session and to have an idea of the structure of the session. I had not done engagement work with young people before so was unsure about how it would all go, but you helped to put my mind at ease and feel really prepared for the session.

(Researcher feedback form)

[names redacted] were fantastic in meeting with us and supporting us in how best to engage with children and young people, having this guidance was very much appreciated.

(Researcher feedback form)

Researchers also mentioned that building relationships with children and young people was important to help facilitate useful discussions:

I think it's really important to have that trusting relationship with the group members that you feel they can tell you if you haven't done something quite right.

(Researcher)

Getting there early was helpful as a way to connect with the young people before the discussion.

(Researcher feedback form)

However, researchers seemed to place less emphasis on using the YPAG to help to generate ideas, and this was only mentioned by one researcher:

We want to design a way of working meaningfully with young people. We felt that the first step we should take was to talk to young people themselves, so that we could hear their ideas about how we could do that.

(Researcher feedback form)

During the focus group, when asked to describe their ideal YPAG session using a blank meeting template with timings, prompts and examples of previous activities, YPAG members described enjoying creative and engaging activities:

I really liked the more creative projects and also the Christmas parties.

(YPAG member)

Suggestions included ‘[Researchers] need to use icebreakers, games, quizzes etc.’ (YPAG member).

This was also true for online meetings. Suggestions included examples of games to play as well as making time for socialising or ‘doing our own thing’. YPAG members said that they found it more difficult to stay engaged with researcher sessions when they are not actively participating.

Sometimes if the researcher's powerpoint isn't very engaging with less opportunities to contribute/do activities, that can be boring.

(YPAG member)

## 5 Discussion

Whilst there has been an increasing recognition of children and young people's involvement in health and care research in recent years [7, 43], there is limited published research evaluating this involvement [32]. Evaluation is important for the ongoing monitoring and development of involvement groups, such as YPAGs, as well as providing opportunities for learning and to identify good practice [31]. This evaluation contributes to filling this evidence gap.

The purpose of undertaking an evaluation of the Herts YPAG was to explore what the group has achieved and how it can be improved for the future. The findings demonstrate that researchers and young people shared the same values of the importance of listening to the voices of children and young people in shaping health and social care research. We also found that being a member of the Herts YPAG has enabled members to build new skills, develop their confidence and provide social opportunities. Some challenges were also highlighted in terms of tokenism and the administrative burden for group facilitators. Conducting this evaluation has provided helpful learning in planning for the group in the future, such as ensuring meetings are creative and engaging, but also ensuring group facilitators provide ways that researchers can provide feedback to the group on how their ideas have shaped their research projects.

### 5.1 Learning From This Evaluation

In this discussion, we consider three key learnings from this evaluation: the importance of meaningful involvement of young people; the potential for creative methods, and some challenges of co‐production.

#### 5.1.1 Meaningful Involvement as a Two‐Way Process

As discussed in the introduction, there is a growing body of knowledge that demonstrates the importance and impact of public involvement in health and care research across the United Kingdom [2, 3, 8, 9] and internationally [5, 6]. The findings of our evaluation build on this evidence base by highlighting the impact and value of YPAGs for both health and care research and the children and young people involved [11, 12, 16].

For involvement to be both effective and uphold a rights‐based approach, it must be a two‐way process which is meaningful, authentic, ongoing, not tokenistic and addresses all elements of the Lundy [28] model: space, voice, audience and influence. As our evaluation shows, involvement in the Herts YPAG not only provides a platform for researchers to seek input into their research projects from children and young people in a convenient way. Membership of the YPAG is also valuable for the development of young people in gaining valuable skills and experience for the future. As we describe above in our findings, young people and researchers both highlighted the importance of the non‐research focused social relationship‐building in generating trust between YPAG members, researchers and creating a community among the YPAG members. This, we argue, is essential in facilitating both space and voice, and thus enabling meaningful involvement.

Ensuring meaningful involvement goes beyond providing fora for young people to express their ideas. It also requires approaches that: seek to build the skills and knowledge of YPAG members; facilitate trust‐building between members and researchers, and enable YPAG facilitators and members to co‐create accessible and inclusive spaces. Providing the opportunity for young people to develop useful skills and improve their confidence and self‐esteem is a clear value of YPAGs [10, 11, 16]. Recognising and reinforcing this value as both essential for, and a positive outcome of, public involvement for children and young people is—we argue—a key element of ensuring involvement is meaningful.

#### 5.1.2 Creative Methods and Co‐Production

Creative methods are well‐suited to co‐produced research, especially with children and young people [23]. However, there is a lack of research on creative methods in public involvement, and there is often confusion between the use of creative methods to facilitate participation and the use of qualitative methodology [25, 26]. As prior research has suggested, use of creative activities fostered connection, interest and engagement from YPAG members. This both led to the generation of more useful ideas for the intended research project and helped to reduce the power imbalance between different members of the ‘Excellent Evaluators’ team.

From the outset, we sought to design and carry out this evaluation collaboratively with YPAG members. Together, the Excellent Evaluators team worked outside of regular YPAG meetings to co‐design the project and co‐deliver data collection and data analysis. Within this article, we have provided an in‐depth account of our co‐working design and show how our co‐production processes were enabled through the adoption and practice of a way of working that combined reflexivity, flexibility and creativity with appropriate resourcing. Further details can be found in our full published evaluation report [33].

This is in line with prior research that shows that creative methods can help foster engagement and interactivity [23]. As Coad [23] further notes, however, such methods are also more time‐consuming. Access to resources such as time, money and skilled facilitation are important requirements for involving young people in health and care research, and YPAGs may be hindered by limited resources and logistical challenges [44]. The capacity of the Herts YPAG to conduct this creative and co‐produced evaluation was enabled because it was valued by the wider research centre in which the Herts YPAG is based. This institutional support was necessary to ensure that dedicated time, resources and staff were allocated to this project.

#### 5.1.3 The Challenges of Co‐Production: The Academic Writing Model

Despite the supportive framework for co‐produced and creative methods, and similar to the eyeYPAG evaluation, it was not possible to achieve full co‐production throughout [16, 20]. During the project design, data collection and data analysis processes, the YPAG members of the Excellent Evaluators team were involved in all aspects and took leadership dependent on capacity and interests. This included a particular focus on working together on data analysis, which has sometimes been considered particularly challenging in co‐production, due to its complexity and time‐consuming nature [22, 45, 46]. By adopting flexible, mixed and creative methods, data analysis was largely conducted collaboratively. However, when finalising the data analysis and writing up for publication, it was agreed within the Excellent Evaluators team that the lead and second author (adults) should take responsibility for this role due to the fluctuating capacity of the young co‐evaluators as secondary school students (revising and exams).

This reflects the power and knowledge differentials between the adult and young members of the Excellent Evaluators team and highlights the concern that—even within an approach that seeks to be co‐produced—how and when children and young people are involved in research is still largely determined and controlled by adults [14]. This also speaks to a wider access issue of conventional approaches to sharing knowledge, in that writing or reading an academic journal article is not accessible (or interesting) to young people. However, whilst we acknowledge that this is a limitation of our co‐produced approach, we have sought to overcome this by producing additional, more accessible materials for sharing the research findings. This includes working with the young members of the Excellent Evaluators team to design the evaluation 2‐page summary. Finally, our blog [36], of which an extract is provided below, sought to provide a more accessible process for the young researchers to reflect on their experience of conducting this evaluation.

#### 5.1.4 Recommendations From the Evaluation

As a direct impact of conducting this evaluation, we have used the feedback from YPAG members and researchers who have worked with the YPAG, to update our ‘Guide to working with the Herts YPAG’, which is provided to all researchers when they are making their session plans to work with the group. This guidance and support aim to ensure that researchers' plans are creative, engaging, accessible and age‐appropriate, making the meetings work better for the YPAG and producing more useful input and impact for the researchers. In addition, we are also exploring how we can increase the capacity of the Herts YPAG through more regular meetings. However, this requires additional staff and YPAG time and capacity. Finally, a key recommendation of this evaluation, as outlined in our report, is to explore in the future how to collect data on tangible medium and longer term impacts of young people's involvement. This would help the YPAG facilitators and members to understand how the YPAG has contributed to research. Together, we aim to regularly review this impact data, and to share our findings with the wider PPI and research community, to continue contributing to the conversation around how to ensure the voice of young people is both heard, and responded to, within research.

### 5.2 Limitations

This was a small study with a limited and self‐selected sample, carried out within a short time frame. All YPAG members were able to participate in the evaluation in one way (either via the in‐person focus group or via the online survey) although the online survey software did not have the same creative activities and therefore may have limited their opportunities for reflection and expression. This was a co‐produced evaluation in which young people and adult facilitators were involved in both conducting and participating in the evaluation. Consequently, the adult facilitators who participated in the second focus group were also involved in analysing and interpreting data relating to their own experiences. This approach differs from the objectivity that may be sought through an independent, formal evaluation. However, it is consistent with our reflective methodology. The participatory, insider approach that we have described in this article was intentionally adopted to align with the values of co‐production, shared decision‐making and meaningful involvement that underpin the YPAG. Our aim is not to draw conclusions from this small evaluation that may directly apply to other YPAGs or involvement groups. Rather, we hope that this study encourages other groups to undertake evaluation activities and provides useful examples of how this may be approached.

### 5.3 Reflections From the Excellent Evaluators Young Researchers

Following the evaluation, in July 2025, the Excellent Evaluators team completed a reflective exercise to consider what they have learnt. Full reflections can be found in the Excellent Evaluators team blog post [36].

- **Jacob:** Leading the focus group, I feel like this really helped with my research skills and my bonds within the YPAG.
- **Benita:** Being able to work in a group as a very independent person and knowing that I'm going to make a change.
- **Zuyi:** Being able to set up and host an in‐person YPAG meeting, despite being a very new member. It's something I thought I would find very challenging, but due to support from Helen and Lauren, I managed to accomplish it, so I am quite proud of what I've done being part of the YPAG so far.

- **Benita:** Understanding some of the material and asking for help. Lauren and Helen have the attitude that no questions are silly, and it really helps with our confidence and asking questions.
- **Jacob:** The whole new research techniques were so hard but all I had to do was ask and it helped me so much.
- **Zuyi:** Sometimes during the focus group it was quiet and hard to gather opinions from everyone, and during the analysis afterwards, it was difficult to interpret what people have said—a sense of ambiguity.

## 6 Conclusion

This evaluation contributes to the evidence for the importance of evaluating children and young people's involvement in research. It also provides useful learning for the Herts YPAG as it continues to develop. The evaluation itself helped to improve the young co‐evaluators understanding of research and build YPAG members' capacity to carry out data collection and analysis. It has also contributed to further developing positive, reflective and meaningful relationships between YPAG members and the staff facilitators. The process of YPAG members being asked for their views and being involved in our work to adapt and respond to these views has, we believe, helped the YPAG itself to meet the four aspects of Lundy's model: space, voice, audience and influence. Given the challenges of the traditional academic knowledge dissemination processes described above, we are pleased to have been awarded additional funding (with thanks to the University of Hertfordshire Economic and Social Research Council Impact Acceleration Account) to co‐produce three additional young people‐focused research outputs with YPAG members: a podcast series; an online zine, and an in‐person event.

As we note at the start of our introduction, there are a range of benefits to the involvement of young people in research: to help improve the quality and impact of research; to support the rights of young people to be involved in research, and to provide young people with a voice in research that receives public funding. This evaluation adds further evidence to demonstrate that YPAGs can provide a means of reaching all three of these goals, if they are appropriately supported and resourced, and when researchers engage with YPAGs with authenticity. Our paper also demonstrates the importance of in‐depth and co‐produced evaluation of this work. We hope it provides encouragement, learning and tools for others seeking to engage in similar reflective processes.

## Author Contributions

**Lauren Denyer:** conceptualization, data curation, formal analysis, visualization, project administration, writing – original draft, writing – review and editing, methodology. **Helen Abnett:** conceptualization, methodology, data curation, investigation, formal analysis, visualization, project administration, writing – original draft, writing – review and editing. **Benita Dijan:** conceptualization, formal analysis, data curation, investigation, writing – review and editing. **Zuyi Su:** conceptualization, methodology, formal analysis, writing – review and editing, visualization. **Jacob Wright:** conceptualization, formal analysis, investigation, methodology, writing – review and editing. **Louca‐Mai Brady:** conceptualization, writing – review and editing, writing – original draft, funding acquisition.

## Ethics Statement

This study received ethical approval from the University of Hertfordshire Health, Science, Engineering and Technology Ethics Committee with Delegated Authority, Protocol number 1007 2025 May HSET. All participants were provided with information about the research and gave their consent (or, for those under 16, asset with their parents or carers providing consent) to participate in the research.

## Conflicts of Interest

The authors declare no conflicts of interest.

## Supporting information


Supporting File

## Data Availability

Research data are not shared.

## References

[1] NIHR , Briefing Notes for Researchers—Public Involvement in NHS, Health and Social Care Research, 2021, https://www.nihr.ac.uk/briefing-notes-researchers-public-involvement-nhs-health-and-social-care-research.

[2] K. Staley , “‘Is It Worth Doing?’ Measuring the Impact of Patient and Public Involvement in Research,” Research Involvement and Engagement 1, no. 1 (2015): 6, 10.1186/s40900‐015‐0008‐5. PMC5598089 29062495

[3] S. Staniszewska , S. Denegri , R. Matthews , and V. Minogue , “Reviewing Progress in Public Involvement in NIHR Research: Developing and Implementing a New Vision for the Future,” BMJ Open 8, no. 7 (2018): e017124, 10.1136/bmjopen‐2017‐017124. PMC6067369 30061427

[4] A. Wearn , K. Brennan‐Tovey , E. A. Adams , et al., “Evaluating Process and Outcomes of Public Involvement in Applied Health and Social Care Research: A Rapid Systematic Review,” Health Expectations 28, no. 1 (2025): e70160, 10.1111/hex.70160. PMC11751718 39840654

[5] S. Gaillard , S. Malik , J. Preston , et al., “Involving Children and Young People in Clinical Research Through the Forum of a European Young Persons' Advisory Group: Needs and Challenges,” Fundamental & Clinical Pharmacology 32, no. 4 (2018): 357–362, 10.1111/fcp.12360. 29457267

[6] T. Richards , Patient and Public Involvement in Research Goes Global, 2017, https://blogs.bmj.com/bmj/2017/11/30/tessa-richards-patient-and-public-involvement-in-research-goes-global/.

[7] L.‐M. Brady and J. Preston , “How Do We Know What Works? Evaluating Data on the Extent and Impact of Young People's Involvement in English Health Research,” Research for All 4, no. 2 (2020): 194–206, 10.14324/rfa.04.2.05.

[8] A. Rouncefield‐Swales , J. Harris , B. Carter , L. Bray , T. Bewley , and R. Martin , “Children and Young People's Contributions to Public Involvement and Engagement Activities in Health‐Related Research: A Scoping Review,” PLoS One 16, no. 6 June (2021): 0252774, 10.1371/journal.pone.0252774. PMC8189547 34106978

[9] A. Warraitch , C. Wacker , S. Biju , et al., “Positive Impacts of Adolescent Involvement in Health Research: An Umbrella Review,” Journal of Adolescent Health 75, no. 2 (2024): 218–230, 10.1016/j.jadohealth.2024.02.029. 38597838

[10] L. Mawn , P. Welsh , L. Kirkpatrick , L. A. D. Webster , and H. J. Stain , “Getting It Right! Enhancing Youth Involvement in Mental Health Research,” Health Expectations 19, no. 4 (2016): 908–919, 10.1111/hex.12386. PMC5152725 26202658

[11] C. Thomas , E. Cockcroft , G. Jenkins , and K. Liabo , “Working With Children and Young People in Research: Supportive Practices and Pathways to Impact,” Journal of Child Health Care 29, no. 1 (2025): 34–52, 10.1177/13674935231171451. PMC11874604 37186542

[12] K. A. Wyatt , J. Bell , J. Cooper , et al., “Involvement of Children and Young People in the Conduct of Health Research: A Rapid Umbrella Review,” Health Expectations 27, no. 3 (2024): e14081, 10.1111/hex.14081. PMC11156690 38845155

[13] UN . Convention on the Rights of the Child, 1989, https://www.ohchr.org/en/instruments-mechanisms/instruments/convention-rights-child.

[14] P. McCafferty and E. Mercado García , “Children's Participation in Social Work Research. A Secondary Data Analysis of an Umbrella Review of Children's Participation in Child Welfare,” British Journal of Social Work 55, no. 4 (2025): 1574–1597, 10.1093/bjsw/bcaf017.

[15] A. Warraitch , M. Lee , D. Bruce , et al., “An Umbrella Review of Reviews on Challenges to Meaningful Adolescent Involvement in Health Research,” Health Expectations 27, no. 1 (2024): e13980, 10.1111/hex.13980. PMC10821743 39102665

[16] L. M. Brady , J. Miller , E. McFarlane‐Rose , J. Noor , R. Noor , and A. Dahlmann‐Noor , “‘We Know That Our Voices Are Valued, and That People Are Actually Going to Listen’: Co‐Producing an Evaluation of a Young People's Research Advisory Group,” Research Involvement and Engagement 9, no. 1 (2023): 1–15, 10.1186/s40900‐023‐00419‐4. PMC10025782 36941695

[17] NIHR , Involving Children and Young People as Advisors in Research, 2024, https://www.learningforinvolvement.org.uk/content/resource/nihr-involving-children-and-young-people-as-advisors-in-research/.

[18] Social Care Institute for Excellence , Co‐Production: What It Is and How to Do It, 2022, https://www.scie.org.uk/co-production/what-how/.

[19] C. Dingelstad , N. Hodyl , R. Wyse , S. Lewis , A. Koschel , and N. Goodwin , “Co‐Production, Co‐Creation and Co‐Design in Primary Health Care: A Scoping Review of Design, Implementation, Impact and Sustainability,” Health Expectations 28, no. 5 (2025): e70447, 10.1111/hex.70447. PMC12818046 41045116

[20] B. Mayall , Children's Childhoods: Observed and Experienced (Psychology Press, 1994).

[21] C. Bradbury‐Jones and J. Taylor , “Engaging With Children as Co‐Researchers: Challenges, Counter‐Challenges and Solutions,” International Journal of Social Research Methodology 18, no. 2 (2015): 161–173, 10.1080/13645579.2013.864589.

[22] J. Coad and R. Evans , “Reflections on Practical Approaches to Involving Children and Young People in the Data Analysis Process,” Children & Society 22, no. 1 (2008): 41–52, 10.1111/j.1099‐0860.2006.00062.x.

[23] J. Coad , “Using Art‐Based Techniques in Engaging Children and Young People in Health CareConsultations and/or Research,” Journal of Research in Nursing 12, no. 5 (2007): 487–497.

[24] J. Leigh , “Using Creative Research Methods and Movement to Encourage Reflection in Children,” Journal of Early Childhood Research 18, no. 2 (2020): 130–142, 10.1177/1476718X19885992.

[25] R. Pandya‐Wood , D. S. Barron , and J. Elliott , “A Framework for Public Involvement at the Design Stage of NHS Health and Social Care Research: Time to Develop Ethically Conscious Standards,” Research Involvement and Engagement 3, no. 1 (2017): 6, 10.1186/s40900‐017‐0058‐y. PMC5611655 29062531

[26] O. R. Phillips , C. Harries , J. Leonardi‐Bee , et al., “What Are the Strengths and Limitations to Utilising Creative Methods in Public and Patient Involvement in Health and Social Care Research? A Qualitative Systematic Review,” Research Involvement and Engagement 10, no. 1 (2024): 48, 10.1186/s40900‐024‐00580‐4. PMC11092192 38741156

[27] L. J. Phillips , M. B. Christensen‐Strynø , and L. Frølunde , “Arts‐Based Co‐production in ParticipatoryResearch: Harnessing Creativity in the Tension Between Process and Product,” Evidence & Policy: A Journal of Research, Debate and Practice 18, no. 2 (2022): 391–411, 10.1332/174426421X16445103995426.

[28] L. Lundy , L. McEvoy , and B. Byrne , “Working With Young Children as Co‐Researchers: An Approach Informed by the United Nations Convention on the Rights of the Child,” Early Education & Development 22, no. 5 (2011): 714–736, 10.1080/10409289.2011.596463.

[29] A. Delahunt , S. Callanan , S. L. Killeen , C. M. McDonnell , and F. M. McAuliffe , “Including the Child's Voice in Research From a Longitudinal Birth Cohort: Insights From the ROLO Young Person's Advisory Group,” Research Involvement and Engagement 9, no. 1 (2023): 2, 10.1186/s40900‐023‐00411‐y. PMC9910271 36759920

[30] G. Pavarini , J. Lorimer , A. Manzini , E. Goundrey‐Smith , and I. Singh , “Co‐Producing Research With Youth: The NeurOx Young People's Advisory Group Model,” Health Expectations 22, no. 4 (2019): 743–751, 10.1111/hex.12911. PMC6737761 31095837

[31] K. Liabo , K. Boddy , S. Bortoli , et al., “Public Involvement in Health Research: What Does ‘Good’ Look Like in Practice?,” Research Involvement and Engagement 6, no. 1 (2020): 11, 10.1186/s40900‐020‐0183‐x. PMC7110674 32266085

[32] L. H. Dewa , A. Lawrence‐Jones , C. Crandell , et al., “Reflections, Impact and Recommendations of a Co‐Produced Qualitative Study With Young People Who Have Experience of Mental Health Difficulties,” Health Expectations 24, no. S1 (2021): 134–146, 10.1111/hex.13088. PMC8137486 32515538

[33] L. Denyer , H. Abnett , B. Dijan , Z. Su , and J. Wright , The Excellent Evaluators Brilliant Blogpost (2025), https://generationr.org.uk/the-excellent-evaluators-brilliant-blogpost/.

[34] *Herts YPAG*. n.d, https://generationr.org.uk/hertfordshire-ypag/.

[35] F. D. Butterfoss , “Process Evaluation for Community Participation,” Annual Review of Public Health 27, no. 1 (2006): 323–340, 10.1146/annurev.publhealth.27.021405.102207. 16533120

[36] L. Denyer , H. Abnett , B. Dijan , Z. Su , J. Wright , and L. M. Brady , Evaluating the Hertfordshire Young People's Research Advisory Group (2025), https://uhra.herts.ac.uk/id/eprint/26074/. 10.1111/hex.70949 PMC13722805 42963773

[37] A. Guan , T. Cruz , J. Sowell , et al., “Combining Mixed Methods and Community‐Based Participatory Research Approaches to Identify Neighborhood‐Level Needs During the COVID‐19 Pandemic,” Journal of Mixed Methods Research 19, no. 1 (2025): 103–117, 10.1177/15586898231222037.

[38] L. Brady and B. Graham , Young Researchers: Toolkit (2022), https://uhra.herts.ac.uk/id/eprint/12585/.

[39] C. Schneijderberg , O. Wieczorek , and I. Steinhardt , The Handbook of Qualitative and Quantitative Content Analysis: Introduction to Classical, Digital, AI‐Supported, and Automated Data Analysis (Taylor and Francis Ltd, 2025).

[40] V. Braun and V. Clarke , “Using Thematic Analysis in Psychology,” Qualitative Research in Psychology 3, no. 2 (2006): 77–101.

[41] V. Braun and V. Clarke , Successful Qualitative Research: A Practical Guide for Beginners (SAGE Publications Inc, 2013).

[42] V. Braun and V. Clarke , Thematic Analysis: A Practical Guide (SAGE Publications Inc, 2022).

[43] M. E. Palm , D. Evans , S. Staniszewska , et al., “Public Involvement in UK Health and Care Research 1995–2020: Reflections From a Witness Seminar,” Research Involvement and Engagement 10, no. 1 (2024): 65, 10.1186/s40900‐024‐00598‐8. PMC11193893 38909270

[44] B. Lipton , H. Dickinson , J. Bailie , et al., “Collaborating with Young People: Identifying the Barriers and Facilitators in Co‐Designed Research,” Health Expectations 28 (2025): e70308, 10.1111/hex.70308. PMC12117193 40432242

[45] H. Kara , Creative Research Methods in the Social Sciences. A Practical Guide (Policy Press, 2015).

[46] J. Mannion , “Creative Collaborative Data Analysis: Co‐Constructing and Co‐Analysing the Data Together,” in Handbook of Creative Data Analysis (Policy Press, 2024), 372–388.

