# Supplementary material for: ‘The Excellent Evaluators’: Co‐Producing an Evaluation of the Hertfordshire Young People's Research Advisory Group (Herts YPAG)
Source: Health Expect. 2026 Oct 30;29(6):e70949. doi: 10.1111/hex.70949 (PMC13722805; doi:10.1111/hex.70949)
Supplement: Supplementary file 1 [file HEX-29-e70949-s001.docx]

**Appendix**

A. **YPAG Member Focus Group Topic Guide**

Ice-breaker activity as group members enter

1. Write or draw on post-it notes and post on flip-charts:
   a. All the activities you have been involved with during your membership of the YPAG;
   b. Activities you would like to get involved in going forward (either doing similar activities again, or totally new activities)

Fruit bowl icebreaker

Warm-up prompts

2. Please finish the sentence: *The YPAG is…..* (go around the room and complete the sentence; facilitators to write up on flip chart/presentation)
3. If you had to explain to a friend what we do in the group, what would you say?

Impact of the group (1)

4. What have you found fun when being a member of the group?
5. Is there anything you’ve found boring, frustrating or confusing?

The ‘ideal’ YPAG session:

6. Working together, fill in this meeting plan to describe the ideal YPAG session (plan will have timings, prompts, examples of activities etc)
7. How does this ‘ideal’ session differ from YPAG meetings you have been a part of?

Impact of the group (2)

8. If we asked friends or family how being in the group has impacted you, what might they say?
9. What skills or knowledge do you think you have gained from being a part of the group? (provide a blank shield template and ask them to fill it in with words/images/symbols/drawings/pictures that show they things they’ve learned)
10. Do you think your feedback makes a difference to researchers? Why or why not?

Reflections

11. Imagine a new member is joining the group. Take one of these postcards, and write a message on it to help them feel welcome.
12. What would you like to do, if the Herts YPAG could do anything at all? (post-its on a piece of cloud-shaped paper)

Final activity

13. Would you recommend the YPAG to a friend or family member to join or present?
B. **Researcher Interview Topic Guide**

Warm-up question

1. Can you tell us what you remember about the first time you worked with the YPAG? What was that like?

Experience working with the group

2. How do you think your relationship with the YPAG has changed over time?
3. Has your approach to involving young people changed since you started working with us? How?
4. What have you appreciated most about working with the group?
5. Are there any particular challenges you have faced when working with the group?
6. Do you think you have been able to work with the group in an inclusive way?

Impact of the group’s feedback

7. Can you share any changes you’ve made to your research because of the group’s feedback?
8. What have you learned about making collaboration with young people work well?
9. Has your work with the group influenced your wider team, research centre, or school in any way?
10. Has your work with the YPAG this shaped the way you involve other communities in your research? If so, how?

Overall reflections

11. Looking back, what are you proud of about your engagement with the group?
12. What advice would you give another researcher who wants to work with the Herts YPAG?
C. **Parent/Carer Survey Questions**

Warm-up question

1. How did your child/young person first get involved in the YPAG?

Impact of the group

2. What impact has YPAG membership had on your child/young person? Are there specific skills, qualities or experiences that they have gained?
3. Are there any challenges your child/young person has experienced or expressed about being part of the YPAG?
4. How do you think the group fits into your child/young person’s life – socially, emotionally, practically or academically?
5. Has your child/young person’s involvement in the group influenced your own understanding or knowledge of research in health? If so, how?

Going forward

6. What would make the group better for new members joining in the future?
7. Is there anything about the group that could be improved?
D. **Researcher Survey Questions**

Warm-up question

1. Can you tell us about the research that you came to the YPAG to talk about?

Experience working with the group

2. What made you want to involve young people in your research?
3. What was it like working with the YPAG?
4. Can you describe your engagement with the YPAG – what kinds of activities did you do?
5. Did you feel all the YPAG members were able to engage in and access the activities that you were doing?
6. Did anything surprise you about the session with the YPAG?

Impact of the group’s feedback?

7. Did you feel like the YPAG members gave you good ideas that you were able to actually use?

- If you did find the ideas useful, can you give an example of how our feedback helped you or your project? Can you describe any changes you made based on our advice?
- If you didn’t feel like the ideas were useful, can you tell us why not? Is there anything that could have made the experience with the YPAG more useful?

8. Have you talked about this group’s impact in publications, presentations, or funding bids? Can you share details?

Inclusion and accessibility

9. Did you feel comfortable when engaging with the YPAG? Ie did it feel welcoming and like a safe space
10. How did this experience compare to working with other advisors or groups?

Overall reflections

11. If you were telling another researcher about the YPAG, what would you say?
12. Would you like to engage with the YPAG again?
13. Is there anything else you would like to share about working with the YPAG?
